# Dataset on leaching properties of coal ashes from Malaysian coal power plant

**DOI:** 10.1016/j.dib.2020.105843

**Published:** 2020-06-08

**Authors:** Salmia Beddu, Teh Sabariah Binti Abd Manan, Mahyun Mohd Zainoodin, Taimur Khan, Wan Hanna Melini Wan Mohtar, Okta Nurika, Hisyam Jusoh, Saba Yavari, Nur Liyana Mohd Kamal, Abdulnoor A. Ghanim, Siddhartha Pati, Mohd Tajuddin Abdullah

**Affiliations:** a Department of Civil Engineering, Universiti Tenaga Nasional, Jalan Ikram-Uniten, 43000 Kajang, Selangor Darul Ehsan, Malaysia; b Institute of Tropical Biodiversity and Sustainable Development, Universiti Malaysia Terengganu, 21300 Kuala Terengganu, Terengganu, Malaysia; c Department of Civil Engineering, Universiti Tenaga Nasional, Jalan Ikram-Uniten, 43000 Kajang, Selangor Darul Ehsan, Malaysia; d Department of Civil Engineering, Faculty of Engineering, Najran University, P.O Box 1988, King Abdulaziz Road, Najran, Saudi Arabia; e Civil Engineering Department, Faculty of Engineering and Built Environment, Universiti Kebangsaan Malaysia, 43600 Bangi, Selangor, Malaysia; f Faculty of Computing and Digital Technology, HELP University, Jalan Sri Semantan 1, Off, Jalan Semantan, Bukit Damansara, 50490 Kuala Lumpur, Malaysia; g Geo TriTech, No. 17, Persiaran Perdana 15A, Pinji Perdana, 31500 Lahat, Perak, Malaysia; h Civil and Environmental Engineering Department, Universiti Teknologi PETRONAS, 32610 Seri Iskandar, Perak Darul Ridzuan, Malaysia; i Department of Civil Engineering, Universiti Tenaga Nasional, Jalan Ikram-Uniten, 43000 Kajang, Selangor Darul Ehsan, Malaysia; j Department of Civil Engineering, Faculty of Engineering, Najran University, P.O Box 1988, King Abdulaziz Road, Najran, Saudi Arabia; k Institute of Tropical Biodiversity and Sustainable Development, Universiti Malaysia Terengganu, 21300 Kuala Terengganu, Terengganu, Malaysia; l Institute of Tropical Biodiversity and Sustainable Development, Universiti Malaysia Terengganu, 21300 Kuala Terengganu, Terengganu, Malaysia

**Keywords:** Leaching properties, Coal ashes, Power plant, Toxicity characteristic leaching procedure, Synthetic precipitation leaching procedure, Heavy metals

## Abstract

Coal combustion by-products (CCPs) (i.e. fly (FA) and bottom (BA) ashes) generated by power plants contain heavy metals. This research presents leaching properties of coal ashes (FA and BA) collected from Jimah coal-fired power station, Port Dickson, Negeri Sembilan using USEPA standard methods namely toxicity characteristic leaching procedure (TCLP), and synthetic precipitation leaching procedure (SPLP). Heavy metals like lead (Pb), zinc (Zn), copper (Cu) and arsenic (As) were quantified using atomic absorption spectrometer (AAS). The leached of heavy metals fluxes were Cu < Zn < Pb < As. As leached the most whilst indicating of possible contamination from As. Overall, the ranges of leached concentration were adhered to permissible limits of hazardous waste criteria for metal (Pb and As) and industrial effluent (Zn and Cu). The presented data has potential reuse as reference for the coal ash concrete mixed design application in construction industries.

Specifications table

**Table:** 

Subject	Engineering
Specific subject area	Environmental Engineering, Civil and Structural Engineering
Type of data	Table
How data were acquired	Instruments: Atomic Absorption Spectrometer (SHIMADZU, AA-6800).
Data format	Raw data
Parameters for data collection	Concentration of heavy metals such as lead (Pb), zinc (Zn), copper (Cu) and arsenic (As) (mg/L)
Description of data collection	The coal ash samples namely fly and bottom ashes were collected from Malaysian coal-fired power station. Fly ash is fine ash particles collected from electrostatic precipitators or filter fabric baghouses. Meanwhile, bottom ash is coarse, granular particles collected from the furnaces bottom.
Data source location	Institution: Department of Civil Engineering, Universiti Tenaga Nasional City/Town/Region: Jalan Ikram-Uniten, 43000 Kajang, Selangor Darul Ehsan Country: Peninsular Malaysia Latitude and longitude (and GPS coordinates) for collected samples/data: Jimah coal-fired power station, Port Dickson, Negeri Sembilan, Peninsular Malaysia (2°35′40.0"N 101°43′24.5"E).
Data accessibility	Data is provided in the article.

## Value of the data

• The data can be used by civil engineers as reference for the application of concrete mix design containing coal ash in construction industries.
• The leaching properties data will benefit scientific community and public authority on hazardous waste management like coal ashes via a strict monitoring and legislated guidelines.
• The data will give an additional value for further spatial temporal studies on leaching properties of heavy metals infused in concrete mixed designs before application in the construction industries to prevent any environmental impact from the use of CCPs.

## 1 Data description

Coal fired power plant generates mass production of coal combustion by-products such as fly (FA) and bottom (BA) ashes [1]. The leaching of heavy metals fluxes may contribute to adverse environmental and health impacts due to bioaccumulation in soft tissues, toxic at certain level of concentration and period of exposure [[2], [3], [4]–5]. Therefore, the dataset presented leaching properties of four heavy metals namely lead (Pb), zinc (Zn), copper (Cu) and arsenic (As) in coal ashes.

The permissible limit of heavy metals for leaching test is categorized as hazardous waste criteria (mg/L) and hazardous waste limit (mg/L) [6]. Hazardous waste criteria^a^ refers (mg/L) to limits that are allowed in solid waste or soil for disposal in a landfill (i.e. Pb: 100.0 mg/L, and; As: 100 mg/L). The hazardous waste limits^b^ (mg/L) are the limits allowed to leach out of soil or solid waste in a landfill (i.e. Pb: 5.0 mg/L, and; As: 5.0 mg/L). However, limits for Zn and Cu are not available. Therefore, limits for industrial effluents regulations [7] and national standards for drinking water quality by Malaysia government [8] are included in this study. The limits for industrial effluent are Standard A (Pb: 0.1 mg/L; Zn: 1.0 mg/L; Cu: 0.2 mg/L and; As: 0.05 mg/L) and Standard B (Pb: 0.5 mg/L; Zn: 1.0 mg/L; Cu: 1.0 mg/L and; As: 0.01 mg/L) [7]. The concentration of heavy metals leached from coal ashes (FA, BA, and FA+BA) after the acidic leaching tests along with standards for hazardous waste are shown in Table 1respectively.

**Table 1:** Comparison of the leaching test values with aqueous concentration threshold by USEPA and the Malaysian industrial effluent regulations: a. Lead (Pb), (b) Zinc (Zn), (c) Copper (Cu) and (d) Arsenic (As).

Heavy metals in fly ash (FA), bottom ash (BA)			Pb	Zn	Cu	As
Concentration of heavy metals (mg/L)	The toxicity characteristic leaching procedure (TCLP)	FA	0.902 ± 0.01	0.216 ± 0.01	0.028 ± 0.00	15.117 ± 0.23
		BA	0.673 ± 0.01	0.149 ± 0.00	0.027 ± 0.00	14.655 ± 0.29
		FA + BA	1.026 ± 0.01	0.196 ± 0.02	0.033 ± 0.00	18.546 ± 0.03
	The synthetic precipitation leaching procedure (SPLP)	FA	0.908 ± 0.00	0.219 ± 0.00	0.030 ± 0.00	15.227 ± 0.11
		BA	0.683 ± 0.01	0.151 ± 0.00	0.028 ± 0.00	14.636 ± 0.07
		FA + BA	1.012 ± 0.01	0.189 ± 0.03	0.033 ± 0.00	18.605 ± 0.17

Permissible limit for metals		Hazardous waste screening criteria^a^ (mg/L)	100.0	None	None	100.0
		Hazardous waste limit^b^ (mg/L)	5.0	None	None	5.0

Limits for Sewage and industrial effluents^c^		Standard A	0.1	1.0	0.2	0.05
		Standard B	0.5	1.0	1.0	0.1

National standards for drinking water quality (mg/L)^d^			0.05	3.0	1.0	0.01

Descriptions			In compliance with permissible limits for metals^ab^	In compliance with limit for sewage and industrial effluent^c^ and national standards^d^		In compliance with permissible limits for metals^b^

In TCLP test, the leached concentrations of Pb were 0.902 ± 0.01 mg/L (FA), 0.673 ± 0.01 mg/L (BA), and 1.026 ± 0.01 mg/L (FA + BA). The leached Zn concentrations were 0.216 ± 0.01 mg/L (FA), 0.149 ± 0.00 mg/L (BA), and 0.196 ± 0.02 mg/L (FA + BA). The leached Cu concentrations were 0.028 ± 0.00 mg/L (FA), 0.272 ± 0.00 mg/L (BA), and 0.033 ± 0.00 mg/L (FA + BA). The leached As concentrations were 15.117 ± 0.23 mg/L (FA), 14.655 ± 0.29 mg/L (BA), and 18.546 ± 0.03 mg/L (FA + BA).

In SPLP test, the leached concentrations of Pb were 0.908 ± 0.00 mg/L (FA), 0.683 ± 0.01 mg/L (BA), and 1.012 ± 0.01 mg/L (FA + BA). The leached concentrations of Zn were 0.219 ± 0.00 mg/L (FA), 0.151 ± 0.00 mg/L (BA), and 0.189 ± 0.03 mg/L (FA + BA). The leached Cu concentrations were 0.030 ± 0.00 mg/L (FA), 0.028 ± 0.00 mg/L (BA), and 0.033 ± 0.00 mg/L (FA + BA). The leached As concentrations were 15.227 ± 0.11 mg/L (FA), 14.636 ± 0.07 mg/L (BA), and 18.605 ± 0.17 mg/L (FA + BA).

The H^+^ ion in aqueous acidic solution will remove the cations (heavy metals) from their binding sites (FA and BA) causing cations leaching due to the reduction of cation exchange capacity (CEC) [9]. Cation exchange capacity is a capacity of cations to be attached on matrix surfaces [10]. Therefore, concentration of heavy metals in the matrix system were found in both TCLP and SPLP tests.

The dataset on leaching properties of coal ashes presented is essentially important. Overall, the ranges of concentration in FA, BA and FA + BA for Pb complied with permissible limits for metals^ab^: Hazardous waste criteria^a^ (mg/L) and TCLP hazardous waste limits^b^ (mg/L). The ranges of concentration for As complied with permissible limits for metal^a^: Hazardous waste criteria^a^ (mg/L). The concentration for Zn and Cu complied with limit for industrial effluent (Standard A and Standard B) and national drinking water quality standards. The evaluation on leaching of heavy metals (Pb, Zn, Cu, and As) in FA, BA and FA + BA showed that As leached the most whilst indicating of possible contamination from As. The leached of heavy metals fluxes were Cu < Zn < Pb < As. Nevertheless, the leachability of mixed samples of FA+BA was found to be higher than those of pure FA or BA in case of Pb, Cu and As. Therefore, the mixed of these samples may expose threat to the environment. Further investigation to understand the physico-chemical and leaching properties of mixed samples are highly recommended.

## 2 Experimental design, materials, and methods

The leaching properties of coal ashes (FA, BA, and FA + BA, 1:1, v/v of dry weight) was determined via two USEPA standard procedures namely toxicity characteristic leaching procedure (TCLP) and synthetic precipitation leaching procedure (SPLP) [11,12].

The TCLP is to determine the leaching properties of organic and inorganic substance in liquid, solid and multiphase wastes simulating landfill leachate (Method 1311) [11]. Two extraction fluid namely extraction fluid No.1 (5.7 mL glacial acetic acid (CH_3_CH_2_OOH) in 500 mL of deionized water, additional of 64.3 mL of 1 M sodium hydroxide (NaOH) and diluted to 1 L, pH: 4.93 ± 0.05) and extraction fluid No. 2 (5.7 mL CH_3_CH_2_OOH diluted to 1 L with deionized water, pH: 2.88 ± 0.05) were used.

The sample ratio to extraction fluid was equivalent to 1:20 (v/v). Samples (5.0 g of each FA, BA and FA + BA) were transferred into a 500 mL Erlenmeyer flask containing 96.5 mL of deionized water and stirred for 5 min. The extraction fluid No. 1 would be added if observed pH was below 5. If the pH was more than 5.0, 3.5 mL 1N HCl would be added. Next, the solutions were heated to 50 °C for 10 min, left to room temperature with agitation at 30 rpm for 18 h.

The synthetic precipitation leaching procedure (SPLP) is to determine the leaching properties of organic and inorganic compounds in liquids, soils, and wastes simulating acid rain from airborne nitric and sulfuric oxides (Method 1312) [12]. Following the same procedure as TCLP, sulfuric acids were used in the extraction fluid instead of acetic acid (5.7 mL sulfuric acid (H_2_SO_4_) in 500 mL of deionized water, additional of 64.3 mL of 1 M sodium hydroxide (NaOH) and diluted to 1 L, pH: 4.20 ± 0.05).

The solutions (contained FA, BA and FA + BA) were filtered using glass fiber filters (0.7 μm) for Atomic Absorption Spectrometry analysis (SHIMADZU, AA-6800). The furnace method assisted with acethylene gas was used for Pb, Cu, and Zn quantification. The graphite method assisted with acethylene gas was used for As quantification.

## Declaration of Competing Interest

The authors declare that they have no known competing financial interests or personal relationships which have, or could be perceived to have, influenced the work reported in this article.

## Appendix Supplementary materials

Image, application 1
